# Identification of immune-infiltrating cell-related biomarkers in hepatocellular carcinoma based on gene co-expression network analysis

**DOI:** 10.1186/s13000-021-01118-y

**Published:** 2021-07-04

**Authors:** Yinghui Hou, Guizhi Zhang

**Affiliations:** Department of Gastroenterology, The Second People’s Hospital of Liaocheng City, No.306 Jiankang Street, Linqing City, 252600 Shandong Province China

**Keywords:** Hepatocellular carcinoma, CD8^+^ T cells, LCK, Weighted gene co-expression network analysis, Diagnosis and prognosis

## Abstract

**Background:**

Hepatocellular carcinoma (HCC) is often caused by chronic liver infection or inflammation. Searching for potential immunotherapy targets will aid the early diagnosis and treatment of HCC.

**Methods:**

Firstly, detailed HCC data were downloaded from The Cancer Genome Atlas database. GDCRNATools was used for the comprehensive analysis of RNA sequencing data. Subsequently, the CIBERSORT package was used to estimate infiltration scores of 22 types of immune cells in complex samples. Furthermore, hub genes were identified via weighted gene co-expression network analysis (WGCNA) and protein-protein interaction (PPI) network analysis. In addition, multiple databases were used to validate the expression of hub gene in the tumor tissue. Finally, prognostic, diagnostic and immunohistochemical analysis of key hub genes was performed.

**Results:**

In the present study, 9 hub genes were identified using WGCNA and PPI network analysis. Furthermore, the expression levels of 9 genes were positively correlated with the infiltration levels of CD8-positive T (CD8^+^ T) cells. In multiple dataset validations, the expression levels of CCL5, CXCR6, CD3E, and LCK were decreased in cancer tissues. In addition, survival analysis revealed that patients with LCK low expression had a poor survival prognosis (*P* < 0.05). Immunohistochemistry results demonstrated that CCL5, CD3E and LCK were expressed at low levels in HCC cancer tissues.

**Conclusion:**

The identification of CCL5, CXCR6, CD3E and LCK may be helpful in the development of early diagnosis and therapy of HCC. LCK may be a potential prognostic biomarker for immunotherapy for HCC.

**Supplementary Information:**

The online version contains supplementary material available at 10.1186/s13000-021-01118-y.

## Background

Hepatocellular carcinoma (HCC) is a common tumor, accounting for 75–85% of primary liver cancer case, and its incidence is on the rise [1, 2]. HCC is caused by chronic hepatitis virus infection, aflatoxin contamination of food, heavy drinking and other factors [3, 4]. The common therapies for HCC are hepatectomy and liver transplantation and ablative therapies; however, the risk is high and the therapeutic effect is unsatisfactory [5]. In recent years, cancer research has focused on immunodiagnostics and immunotherapy [6, 7].

Previous study has demonstrated that tumor-infiltrating immune cells (TIICs) can help the host resist the development of cancer cells and solid tumors [7]. The density and type of TIICs are closely associated with the clinical outcome of the tumor [8–10]. Among which, CD8-positive T (CD8^+^ T) cells, account for a large proportion of immune cells in a number of cancer types, have been demonstrated to play a key role in controlling tumor progression [11, 12]. In addition, previous study has found that D8^+^CXCR5^+^T cells are highly invasive and well infiltrated, which enables patients with HCC to have an improved prognosis [13]. Loss of the immune-mediated cancer field (ICF) can lead to reduction and shrinkage of liver tumors [14]. The Gene Set Enrichment Analysis (GSEA) analysis revealed that the local immune phenotype of HCC with tumor protein p53 (TP53) mutation was reduced [15]. Programmed cell death protein 1 (PD-1) can induce the immune checkpoint response of T cells and enable tumor cells to evade immune monitoring. Its inhibitor of receptor PD-L1 can effectively inhibit this signaling pathway and improve the therapeutic effect [16]. However, the specific mechanism of their immunotherapy remains unknown. Therefore, exploring biomarkers related to immune infiltration will help detect the HCC immunotherapy response and identify specific immune mechanisms.

With the development of biological information technology, a variety of tools can be used to identify biomarkers. For example, machine learning [17], weighted gene co-expression network analysis (WGCNA) [18] and CIBERSORT [19] have been widely used to search for biomarkers. To explore the role of the microenvironment and identify potential biomarkers for HCC, WGCNA and CIBERSORT were utilized, followed by protein-protein interaction (PPI) network construction, and hub gene validation and identification in the present study.

## Methods

### Data

GDCRNATools, a novel R package, was used for the comprehensive analysis of RNA sequencing (RNA-seq) data [20]. RNA-seq data and clinical information for HCC were downloaded from The Cancer Genome Atlas (TCGA) database (http://cancergenome.nih.gov/) on August 27, 2020. A total of 370 HCC samples with survival data and 50 adjacent non-tumor samples were obtained by removing patients lack of survival information based on clinical information [21]. Genes identified by using RNA expression profiles were annotated based on the Ensembl gene ID. Genes with missing expression values in > 20% of samples or patients and genes with 0 expression values in all samples were excluded. Voom standardization was performed to screen gene expression data. In the present study, all data were obtained from public databases without the approval of an ethics committee.

### Evaluation of TIICs

The “CIBERSORT” package in R was used to estimate infiltration scores of 22 types of immune cells in complex samples [12]. The processed gene expression matrix was uploaded to the CIBERSORT (https://cibersort.stanford.edu/) web tool. LM22 expression signature and 500 permutations were used for the algorithm. Subsequently, the percentages of immune cells for each sample were selected as WGCNA trait data. Wilcoxon signed-rank test and the “ggplot2” package in R (version 3.1.1) were used to compare 22 types of immune cells between groups and for visualization, respectively.

### Co-expression network construction

The “WGCNA” package in R (http://www.r-project.org/) was used for WGCNA analysis of genes (the first 25% of the variation coefficient of gene expression matrix) in HCC samples from TCGA. Firstly, the expression levels of single transcripts were converted into a similarity matrix based on the Pearson’s correlation value between paired genes. Subsequently, the similarity matrix was converted into an adjacency matrix. β = 5 was selected as the soft-thresholding power. When the power of β = 5, the adjacency matrix was converted into a topological overlap matrix. Genes were classified into different modules using the dynamic hybrid cutting method, and the minimum module size cut-off value was 30.

### Identification of hub module

The significance of modules was determined using a Pearson test (*P* < 0.05), which calculate the correlation between module eigengenes and immune infiltrated cells. Subsequently, the differences of module eigengenes were further calculated and visualized. A cutting line for the module tree diagram was selected and some modules were merged. The module most relevant to the immune cells of interest was selected and defined as the hub module. CIBERSORT was used to calculate the infiltration of immune cells of interest in HCC samples and adjacent non-tumor samples in the GSE14520 dataset (comprising 225 tumor samples and 220 adjacent non-tumor samples) and the GSE54236 dataset (comprising 81 tumor samples and 80 adjacent non-tumor samples). The GSE14520 and GSE54236 datasets were obtained from the Gene Expression Omnibus (GEO) database [22].

### Functional analysis of genes in the hub module

To further explore the biological function of genes, the online tool Database for Annotation, Visualization and Integrated Discovery (DAVID) 6.8 (https://david.ncifcrf.gov/) was used for Gene Ontology (GO) and Kyoto Encyclopedia of Genes and Genomics (KEGG) pathway enrichment analyses. The “GOplot” package in R (version 1.0.2) was used for visualization of the enrichment results.

### Identification of hub genes

Candidate hub genes were selected according to the module connectivity (MM) and clinical trait relationship (GS) of each gene in the hub module. Genes with MM > 0.8 and GS > 0.5 in the module were selected as candidate hub genes. Furthermore, the Search Tool for the Retrieval of Interacting Genes/Proteins (STRING) database (https://string-db.org/) was used to construct the PPI network for all genes in the hub module. The confidence between nodes in the PPI network was > 0.7. Subsequently, Cytoscape software (http://www.cytoscape.org) was used for the visualization of the PPI network. Genes with degrees > 25 were considered as central nodes. Online tools were used to perform Venn analysis (http://www.bioinformatics.com.cn/) on candidate hub genes and central nodes in the PPI network.

### Validation of hub genes

Two immune-related databases based on TCGA but different from the CIBERSORT algorithm were used to validate these hub genes. Firstly, Tumor Immune Estimation Resource (TIMER) was used to obtain CD8^+^ T cell content in each HCC sample [23]. The Spearman correlation between CD8^+^ T cell and hub genes was calculated, and the “ggplot2” package in R (version 3.1.1) was used to visualize the results. Subsequently, Tumor Immune System Interactions Database (TISIDB) was used to determine the Spearman correlation between hub genes and TIICs [24]. The heatmap constructed by the “pheatmap” package in R was used to visualize these results.

### Correlation between hub genes and immune factors

The TISIDB database was used to obtain the Spearman correlation between hub genes and immunosuppressive factors, immune-stimulating factors, chemokines and receptors. Subsequently, the “heatmap” package in R was used to construct the heatmap. Hub genes and immune factors with average correlation > 0.5 were selected. Then, String was used to construct the PPI network for these immune factors and hub genes. Cytoscape software was used for the visualization of the PPI network. This will be helpful to explore the infiltration mechanism of CD8^+^ T cell.

### Identification of clinical characteristics of hub genes

The RNA expression data in HCC were acquired from TCGA. The Wilcoxon signed-rank test was used to analyze the statistically significant differences between adjacent non-tumor samples and tumor samples. In addition, the “limma” package in R was used to analyze the differences in all coding genes [25], and the “ggplot2” package in R was used to draw a volcano plot. Then, *P*-value (P) < 0.05 and |log2FoldChange| (|log2FC|) > 1 was used to identify differentially expressed genes. Expression validation of hub genes was performed using the GSE14520 and GSE54236 datasets which were obtained from the GEO database [22]. The protein expression levels of hub genes were verified using the Proteomic Data Commons Database (https://pdc.cancer.gov, comprising 316 tumor samples and 316 adjacent non-tumor samples). Finally, violin diagrams were constructed to demonstrate the correlation between hub genes and clinical stages in TCGA. A Kruskal-Wallis test was used for the analysis of statistical significance.

### Prognosis, diagnostic and immunohistochemical analysis

Gene Expression Profiling Interactive Analysis (GEPIA, http://gepia.cancer-pku.cn/) is an online tool of gene expression analysis, which provides data on gene expression, tumor stage and survival for 33 cancer types, including HCC [26]. This online tool was used to analyze the survival ability of key hub genes. Additionally, TCGA data were used for diagnostic and immunohistochemical analysis of key hub genes. The Human Protein Atlas (https://www.proteinatlas.org/) online analysis software was used to perform immunohistochemical analysis.

## Results

### Infiltration of immune cells

**Fig. 1:**
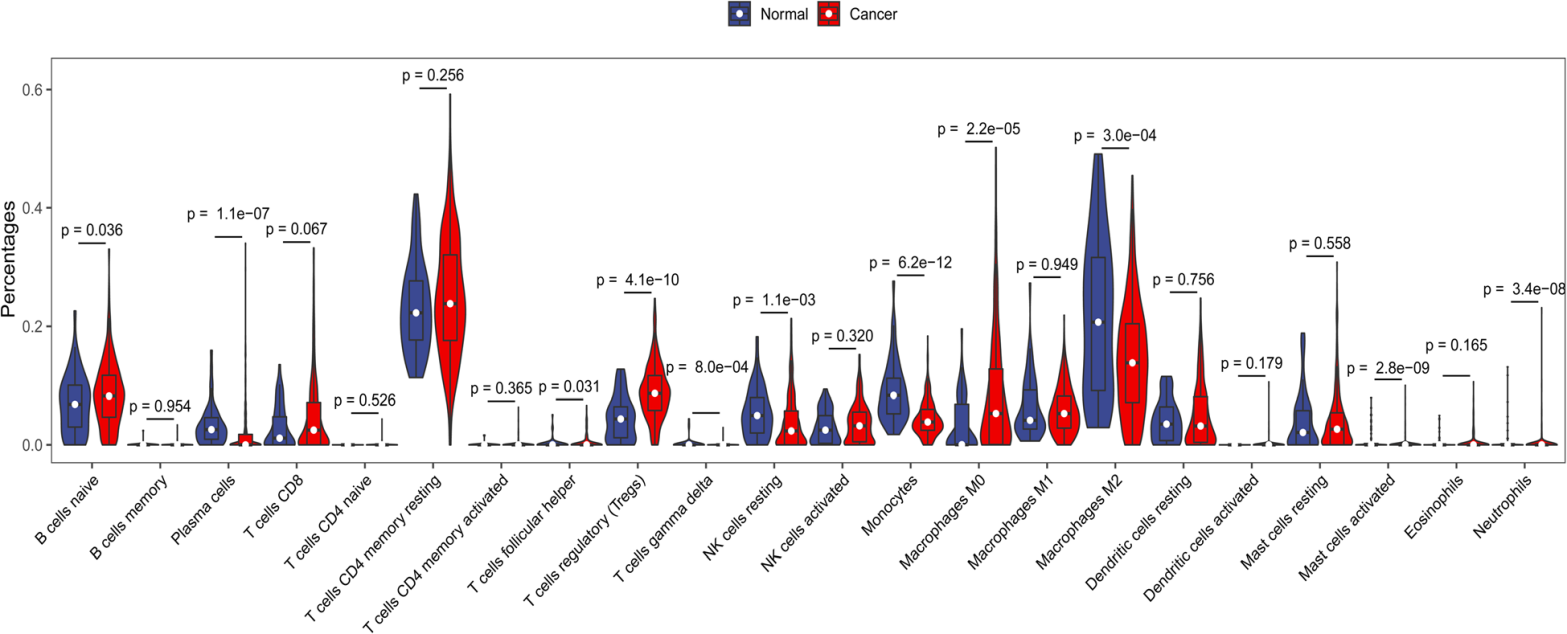
Difference analysis in immune infiltration between the HCC samples and adjacent non-tumor samples. Normal and cancer indicate adjacent non-tumor samples and HCC samples, respectively. *P* < 0.05 was considered to indicate a statistically significant difference. HCC: hepatocellular carcinoma

CIBERSORT calculated the infiltration of immune cells in HCC samples and adjacent non-tumor samples. Compared with adjacent non-tumor samples, the degree of infiltration of B cells naive (*P* = 0.036), T cells regulatory (Tregs) (*P* = 4.1e-10) and Macrophages M0 (*P* = 2.2e-05) in tumor tissues was significantly increased, whereas the degree of infiltration of Plasma cells (*P* = 1.1e-07), NK cells resting (*P* = 1.1e-03), Monocytes (*P* = 6.2e-12) and Macrophages M2 (*P* = 3.0e-04) was significantly decreased (Fig. 1). This indicated that the occurrence and development of HCC are closely associated with immune cells.

### Construction of WGCNA

**Fig. 2:**
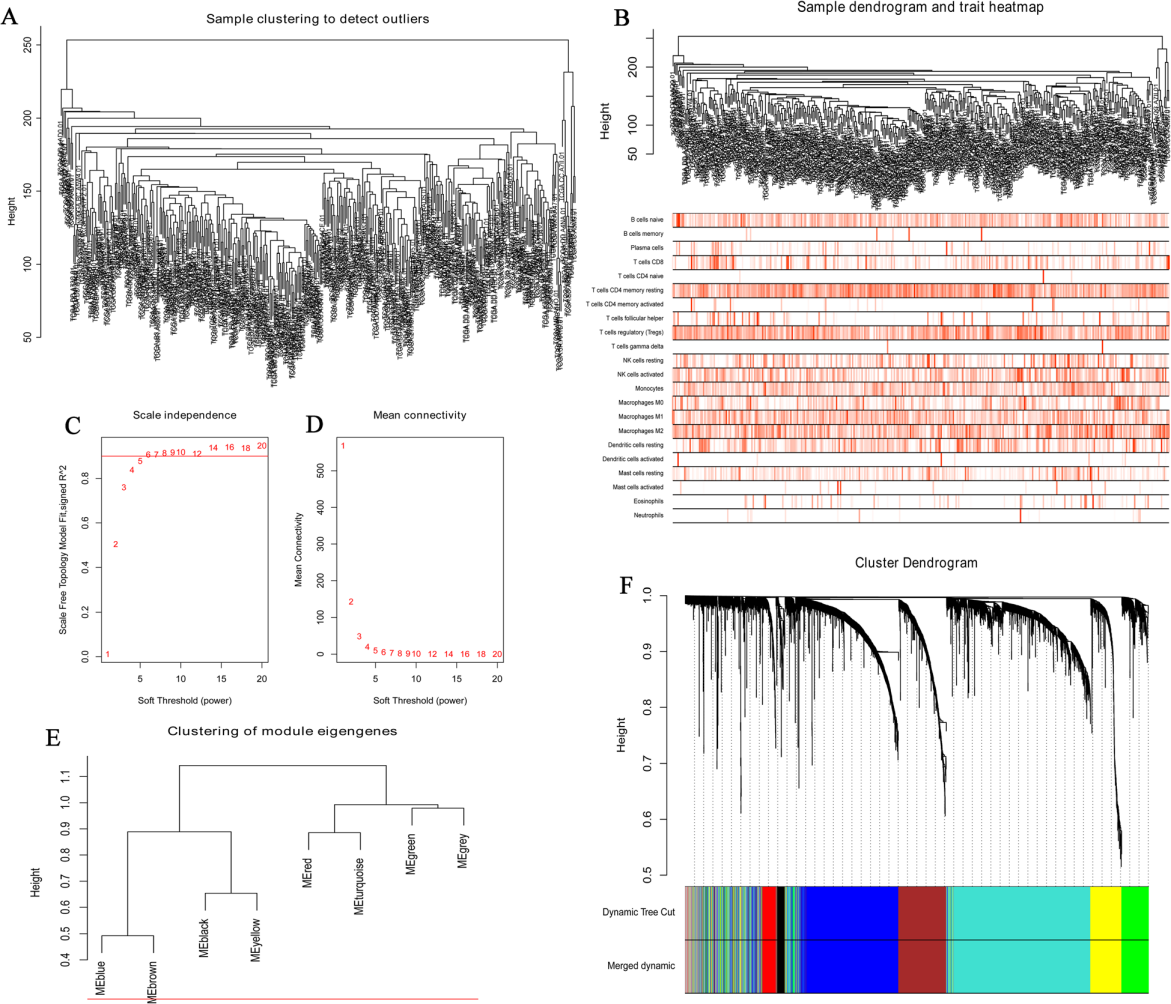
Cluster analysis and construction of WGCNA. **A** Sample clustering. **B** Sample dendrogram and trait heatmap. In the heatmap, the darker the color, the higher the degree of cell infiltration. **C** The scale-free fitting index of different soft threshold power (β). **D** The average connectivity of various soft threshold powers. **E** The horizontal line indicates the module merge threshold. **F** Clustering results of modules in gene data in WGCNA analysis. WGCNA: weighted gene co-expression network analysis

To identify the hub genes, 3750 genes were selected to construct the WGCNA. Firstly, cluster analysis was carried out on the samples and no outliers were found (Fig. 2a). A dendrogram and trait heatmap of 370 samples were constructed (Fig. 2b), and darker color indicates a higher degree of infiltration. When the parameter value of the weight coefficient is 5, it approximates a scale-free topology. β = 5 was regarded as soft-thresholding power to construct a scale-free network (Fig. 2c, d). The dynamic tree cutting method was used to merge the modules with a difference < 25%. Finally, 8 modules were identified (Fig. 2e, f).

### Identification and enrichment analysis of hub module

**Fig. 3:**
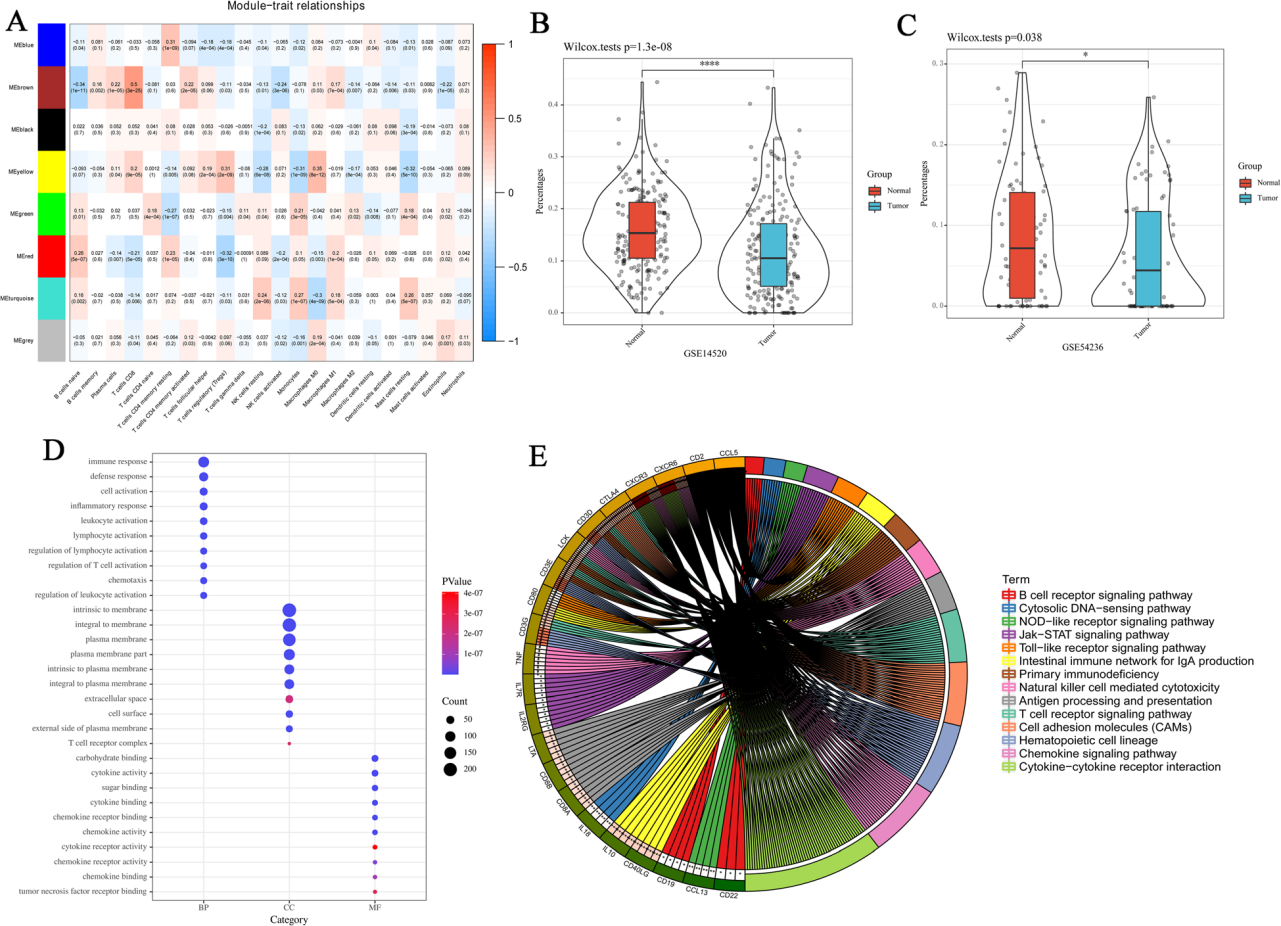
Identification and functional enrichment of hub modules. **A** Heatmap showing associations between the module characteristic genes and immune cell infiltration. **B** CD8^+^ T cell infiltration in HCC samples and adjacent non-tumor samples from the GSE14520 dataset. Normal and Tumor indicate adjacent non-tumor samples and HCC samples, respectively. **C** CD8^+^ T cell infiltration in HCC samples and adjacent non-tumor samples from the GSE54236 data set. Normal and Tumor indicate adjacent non-tumor samples and HCC samples, respectively. **D** Significantly enriched GO terms. GO: Gene Ontology; BP: Biological process; CC: Cell composition; MF: Molecular function. **E** Significantly enriched KEGG pathways. For KEGG analysis, different colors represent different signaling pathways. KEGG, Kyoto Encyclopedia of Genes and Genomes

To determine the hub module, the correlation between the characteristic genes of the module and immune infiltrated cells was calculated using a Pearson test (*P* < 0.05). Eight modules, the brown module was highly related to CD8^+^ T cells (*R*^*2*^ = 0.5, *P* = 3e-25), and the yellow module was related to Macrophages M0 (*R*^*2*^ = 0.35, *P* = 8e-12) (Fig. 3a). The present study particularly focused on CD8^+^ T cells. In TCGA database, no significant difference (*P* = 0.067) in the percentage of CD8^+^ T cells infiltration was observed between HCC samples and adjacent non-tumor samples. Therefore, the immune infiltration of CD8^+^ T cells in HCC samples and adjacent non-tumor samples in GEO was further verified. Compared with adjacent non-tumor samples, the percentage of CD8^+^ T cells infiltration was markedly decreased in HCC samples (Fig. 3b, c).

The present study focused on the brown module related to CD8^+^ T cells and considered it as a hub module. To further explore the biological functions of genes in the hub module, the online tool DAVID 6.8 was used for enrichment analysis. According to GO analysis, most of the genes were distributed on the plasma membrane or membrane surface, and participate in the immune response and the activation of leukocytes, lymphocytes, T cells and other immune cells (Fig. 3d). In addition, based on KEGG analysis, most of the genes were involved in cytokine-cytokine receptor interaction, chemokine signaling pathway, T cell receptor signaling pathway and other signaling pathways related to the immune response (Fig. 3e).

### Identification of hub genes

**Fig. 4:**
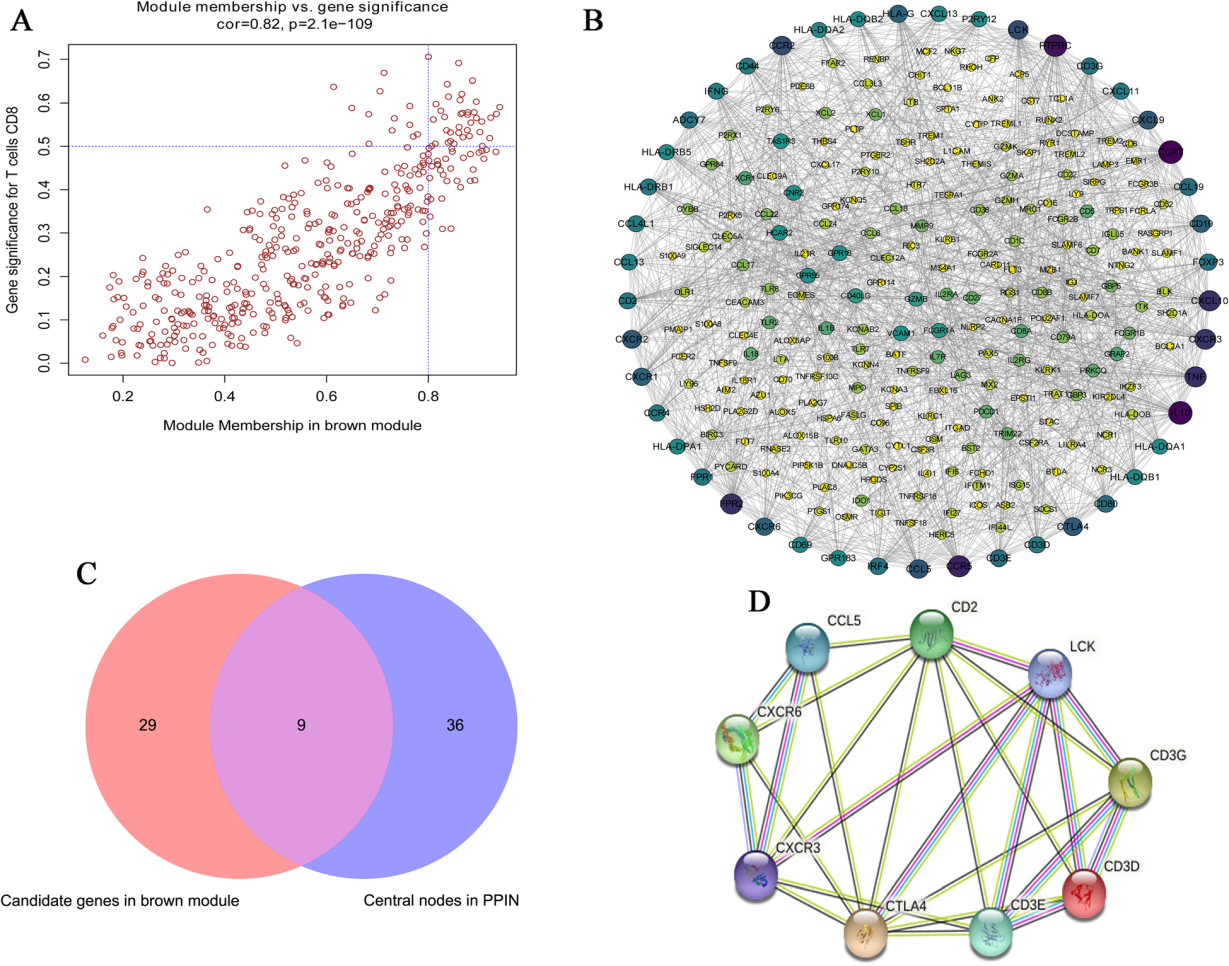
Identification of hub genes. **A** Scatter diagram of genes in brown module. Each red circle represents a gene. **B** PPI network of all genes in the hub module. The outermost circle is the center node. **C** Overlapping genes between the PPI network and scatter diagram (module connectivity > 0.8 and clinical trait relationship > 0.5) were selected as hub genes. **D** PPI network of the 9 hub genes. PPI, protein-protein interaction

After identifying the hub module, the hub genes in the hub module were further explored. Based on cutoff values of MM > 0.8 and GS > 0.5 as cutoff values, 38 genes were selected (Fig. 4a). In the PPI network, 45 central nodes (degree > 25) were screened out (Fig. 4b). In the Venn analysis, 9 intersection genes were screened out (Fig. 4c), and these were considered to be hub genes (CCL5, CD2, CD3D, CD3E, CD3G, CTLA4, CXCR3, CXCR6, LCK). A PPI network was constructed for 9 hub genes (Fig. 4d). The results demonstrated showed that these 9 hub genes were interrelated. This suggested that these 9 hub genes may interact with each other to in the development of HCC.

### Validation of hub genes

**Fig. 5:**
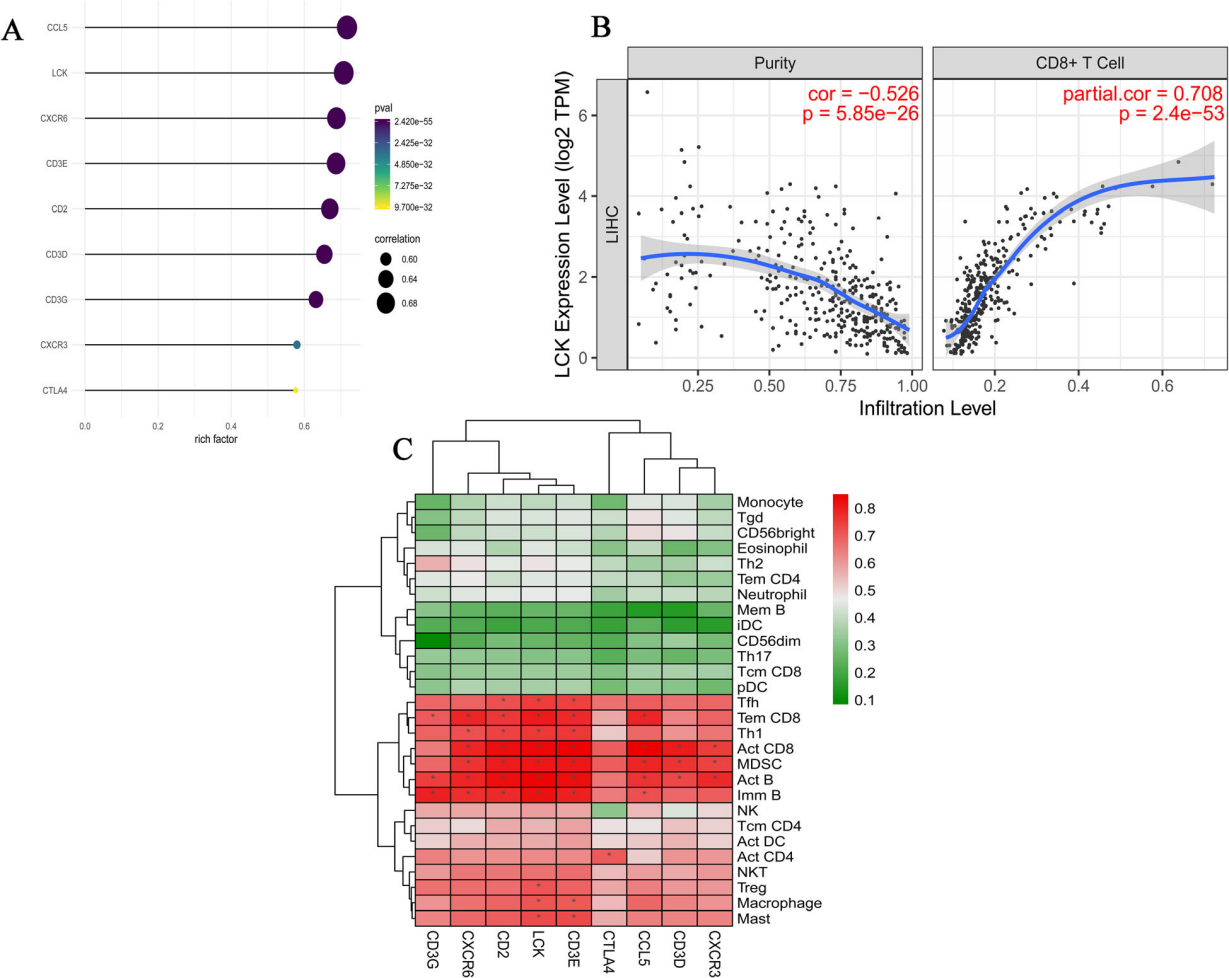
Validation of hub genes. **A** Association between the expression levels of 9 hub genes and CD8^+^ T cell infiltration levels in TIMER database. **B** Association between LCK expression and CD8^+^ T cell infiltration levels in TIMER database. **C** Association between the expression levels of the 9 hub genes and tumor-infiltrating immune cells (TIICs) in TISIDB database. Red indicates a high correlation and green indicates a low correlation. TIMER: Tumor Immune Estimation Resource

To study the relationship between these hub genes and CD8^+^ T cells, the expression data of hub genes in the TIMER database were analyzed. The analysis results revealed that the expression levels of 9 hub genes were positively associated with the infiltration levels of CD8^+^ T cells (Fig. 5a). For example, a correlation scatter plot between LCK expression and the infiltration levels of CD8^+^ T cells is shown (Fig. 5b). Additionally, the association between the abundance of TIICs and hub gene expression were explored. Analysis based on the TISIDB database showed that hub genes were positively associated with numerous TIICs (Fig. 5c).

### Immune and clinical characteristics

**Fig. 6:**
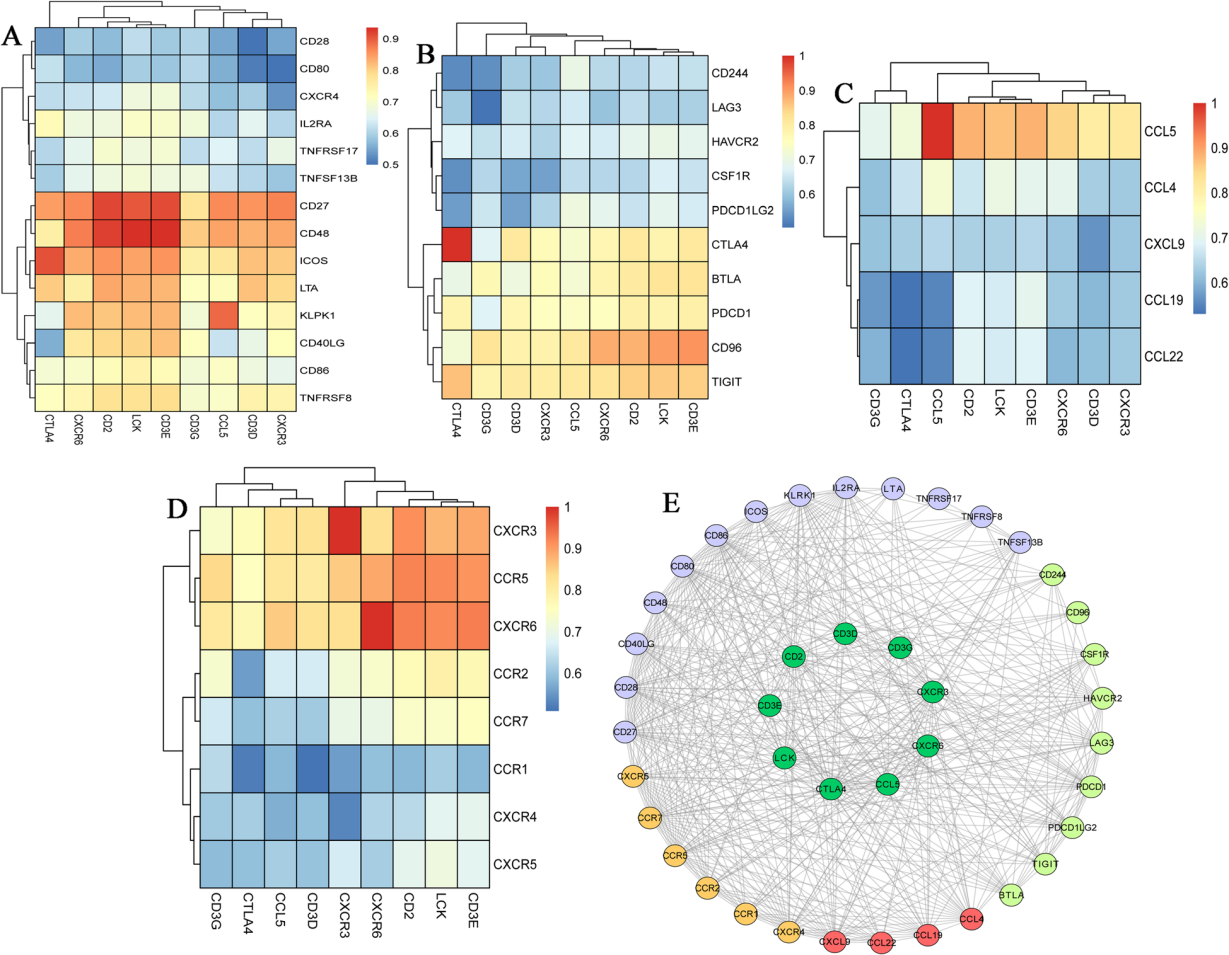
Association between 9 hub genes and immune factors. **A** Heatmap of association between 9 hub genes and immune stimulating factors. **B** Heatmap of association between 9 hub genes and immunosuppressive factors. **C** Heatmap of association between 9 hub genes and chemokines. **D** Heatmap of association between 9 hub genes and receptors. **E** Protein-protein interaction (PPI) network of 9 hub genes and immune factors. The average correlation between 32 immune-related factors and 9 hub genes was > 0.5

**Fig. 7:**
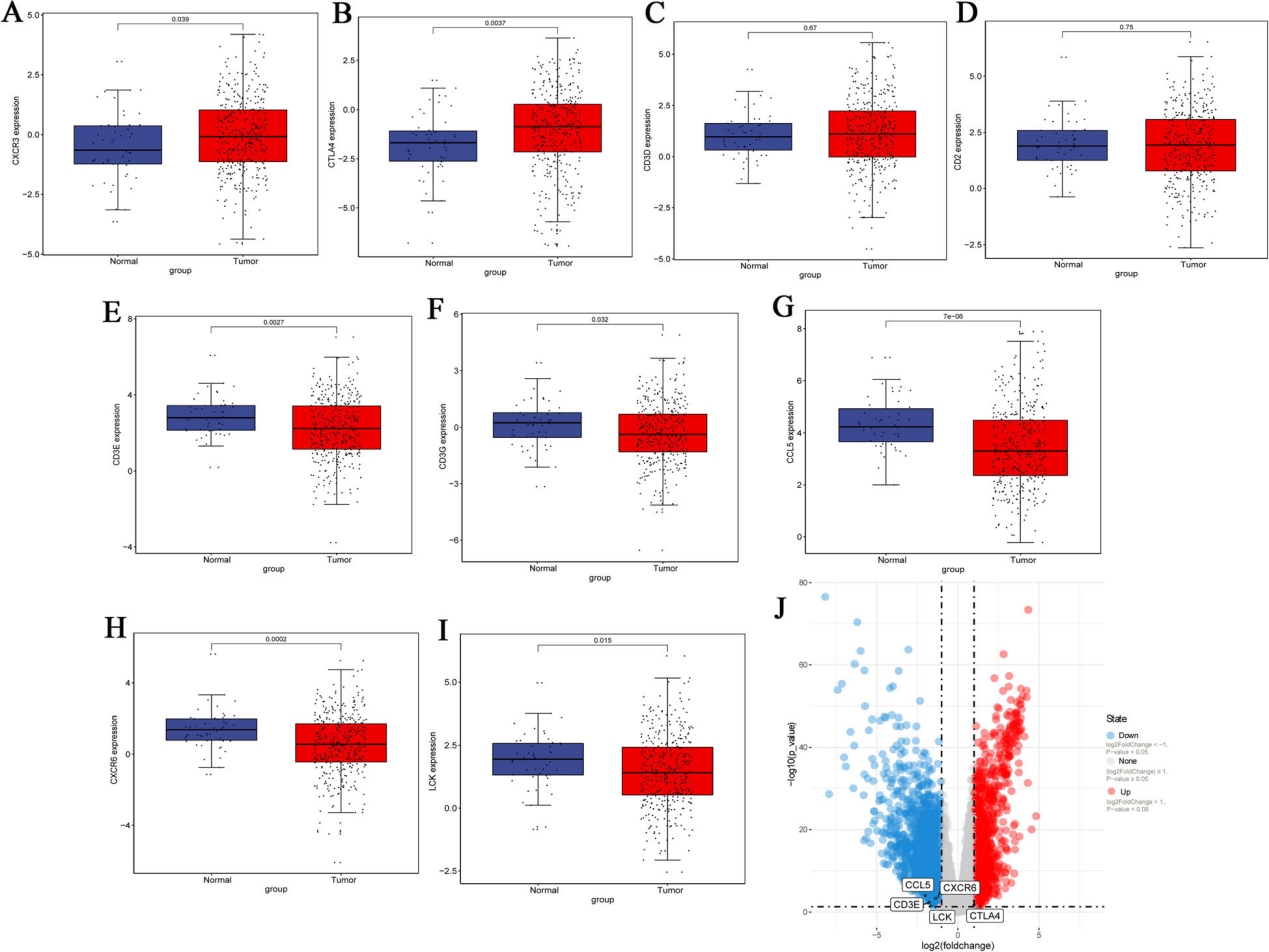
Verification of hub genes expression in HCC samples and adjacent non-tumor samples using the TCGA database. **A**-**I** Differential expression of CXCR3, CTLA4, CD3D, CD2, CD3E, CD3G, CCL5, CXCR6 and LCK in normal and tumor samples. Normal and Tumor indicate adjacent non-tumor samples and HCC samples, respectively. *P* < 0.05 was considered to indicate a statistically significant difference. **J** Volcano plot of differentially expressed genes in the adjacent non-tumor samples and HCC samples. Red dots, blue dots and black circles represent up-regulated genes, down-regulated and hub genes, respectively. *P* < 0.05 and |log2FC| > 1 was used to identify differentially expressed genes. HCC: hepatocellular carcinoma

The Spearman correlation between the expression levels of 9 hub genes and immune factors was searched in the TISIDB database. A total of 14 immune-stimulating factors, 10 immunosuppressive factors, 5 chemokines and 8 receptors were identified (Fig. 6a-d). Among them, the average correlation between the 32 immune-related factors and 9 hub genes was greater than 0.5. The STRING database was used to construct an immune infiltration interaction network to explore the infiltration mechanism of CD8^+^ T cells (Fig. 6e). Expression levels of 9 hub genes in adjacent non-tumor samples and tumor samples were obtained from TCGA. The expression levels of CXCR3 and CTLA4 were higher in tumor tissues than those in normal tissues (*P* < 0.05) (Fig. 7a, b). Compared with normal control tissues, the expression level of CD3D and CD2 in tumor tissues were not significantly different (Fig. 7c, d), while the expression levels of CD3E, CD3G, CCL5, CXCR6 and LCK in tumor tissues were lower than those in normal tissues (*P* < 0.05) (Fig. 7e-i). In addition, the volcano map revealed that the expression levels of CCL5, CXCR6, CD3E and LCK were up-regulated in tumor tissues, and CTLA4 expression was down-regulated in tumor tissues compared with in normal tissues (Fig. 7j) based on screening criteria of *P* < 0.05 and |log2FC| > 1.

**Fig. 8:**
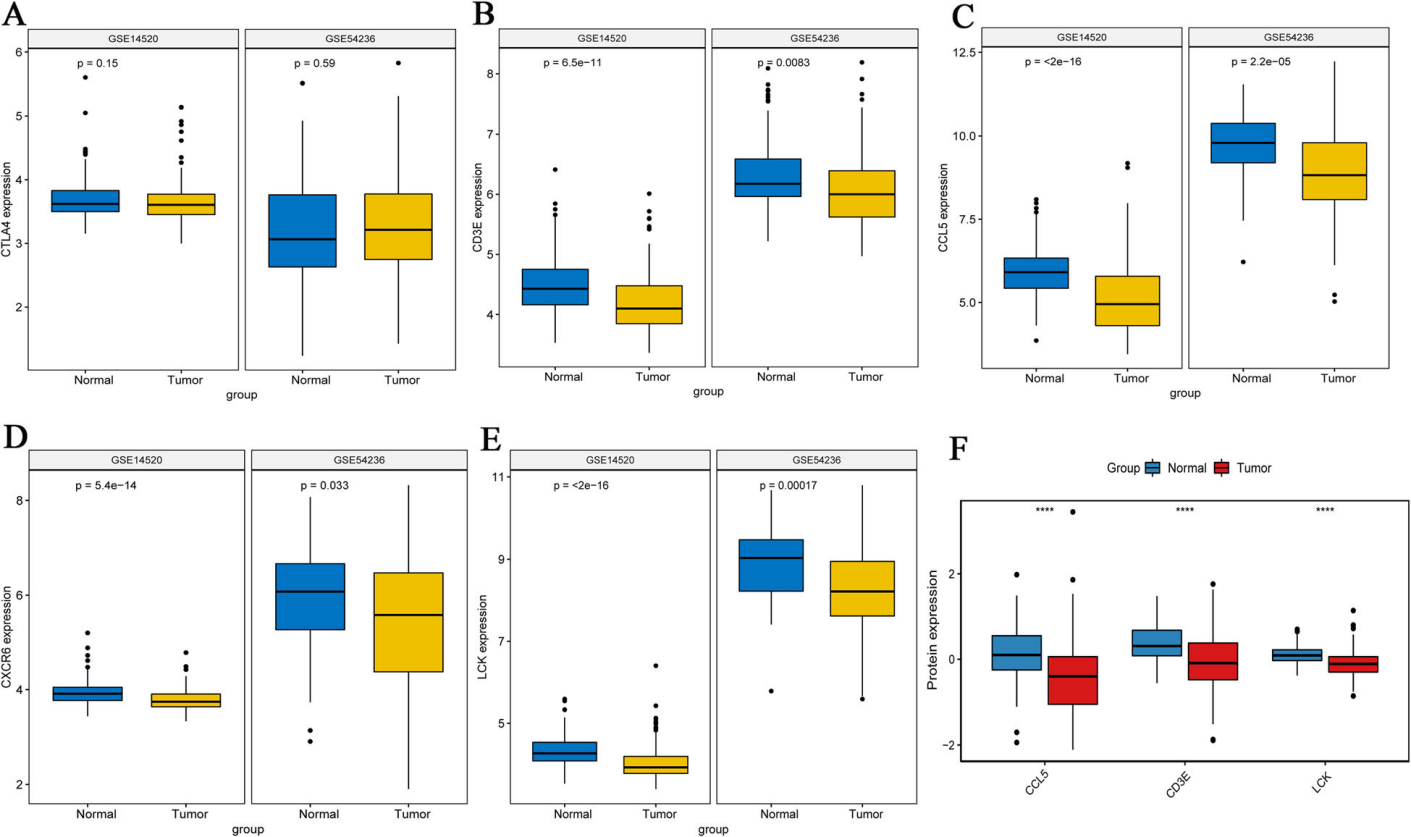
Differential expression of CTLA4, CD3E, CCL5, CXCR6 and LCK in HCC samples and adjacent non-tumor samples. **A**-**E** Differential expression of genes, including CTLA4 (**A**), CD3E (**B**), CCL5 (**C**), CXCR6 (**D**) and LCK (**E**) in HCC samples and adjacent non-tumor samples in the GSE14520 and GSE54236 datasets. **F** Differential expression of proteins, including CD3E, CCL5 and LCK in HCC samples and adjacent non-tumor samples from the Proteomic Data Commons data set. Normal and Tumor indicate adjacent non-tumor samples and HCC samples, respectively. *P* < 0.05 was considered to indicate a statistically significant difference. ****, *P* < 0.0001. HCC: hepatocellular carcinoma

**Fig. 9:**
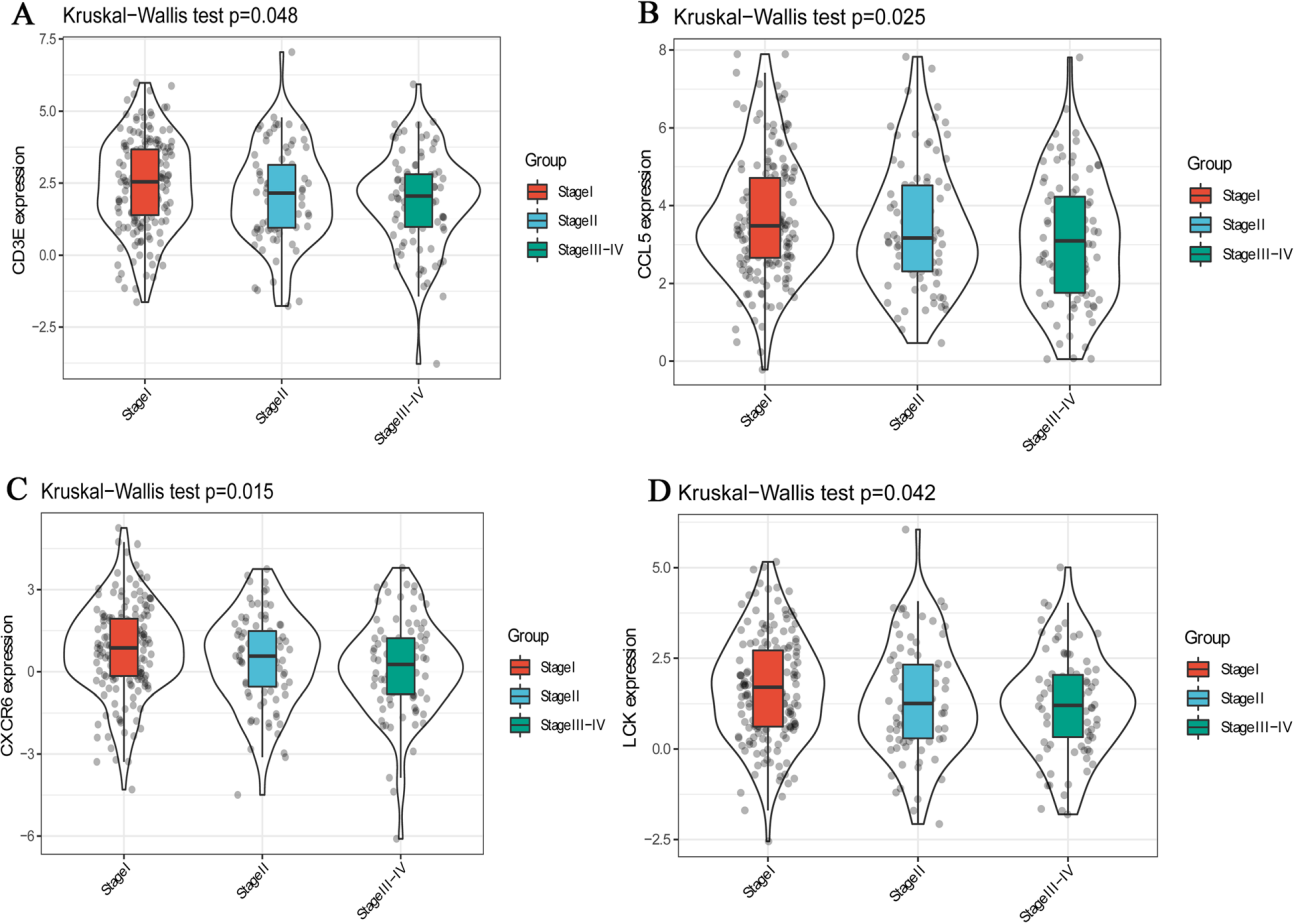
Differential expression of CD3E (**A**), CCL5 (**B**), CXCR6 (**C**) and LCK (**D**) in different pathological stages. *P* < 0.05 was considered to indicate a statistically significant difference. A Kruskal-Wallis test was used to analyze the statistical significance among stage I, stage II and stage III-IV

Further expression verification of these 5 hub genes was performed in GSE14520 and GSE54236. Compared with normal control tissues, the expression level of CTLA4 in tumor tissues was not significantly different (Fig. 8a). Notably, the expression levels of CD3E, CCL5, CXCR6 and LCK in tumor samples and adjacent non-tumor samples were markedly different (Fig. 8b-e), and the results were consistent with the results of the analysis using TCGA. In addition, the protein expression levels of hub genes were further verified using the Proteomic Data Commons Database. It is a pity CXCR6 is not found in the Proteomic Data Commons Database. Therefore, only the protein expression levels of CCL5, CD3E, and LCK were verified. The expression levels of CCL5, CD3E, and LCK were lower in tumor tissues samples than those in adjacent non-tumor tissue samples (Fig. 8f), which is consistent with the results of transcriptomics. The expression levels of these 4 hub genes in different pathological stages and tumor grades of HCC were investigated. In general, gene expression levels decreased with the increase in pathological stage (Fig. 9). However, there was no significant difference between tumor grades (Supplementary Fig. 1).

### Prognosis, diagnostic and immunohistochemical analysis

**Fig. 10:**
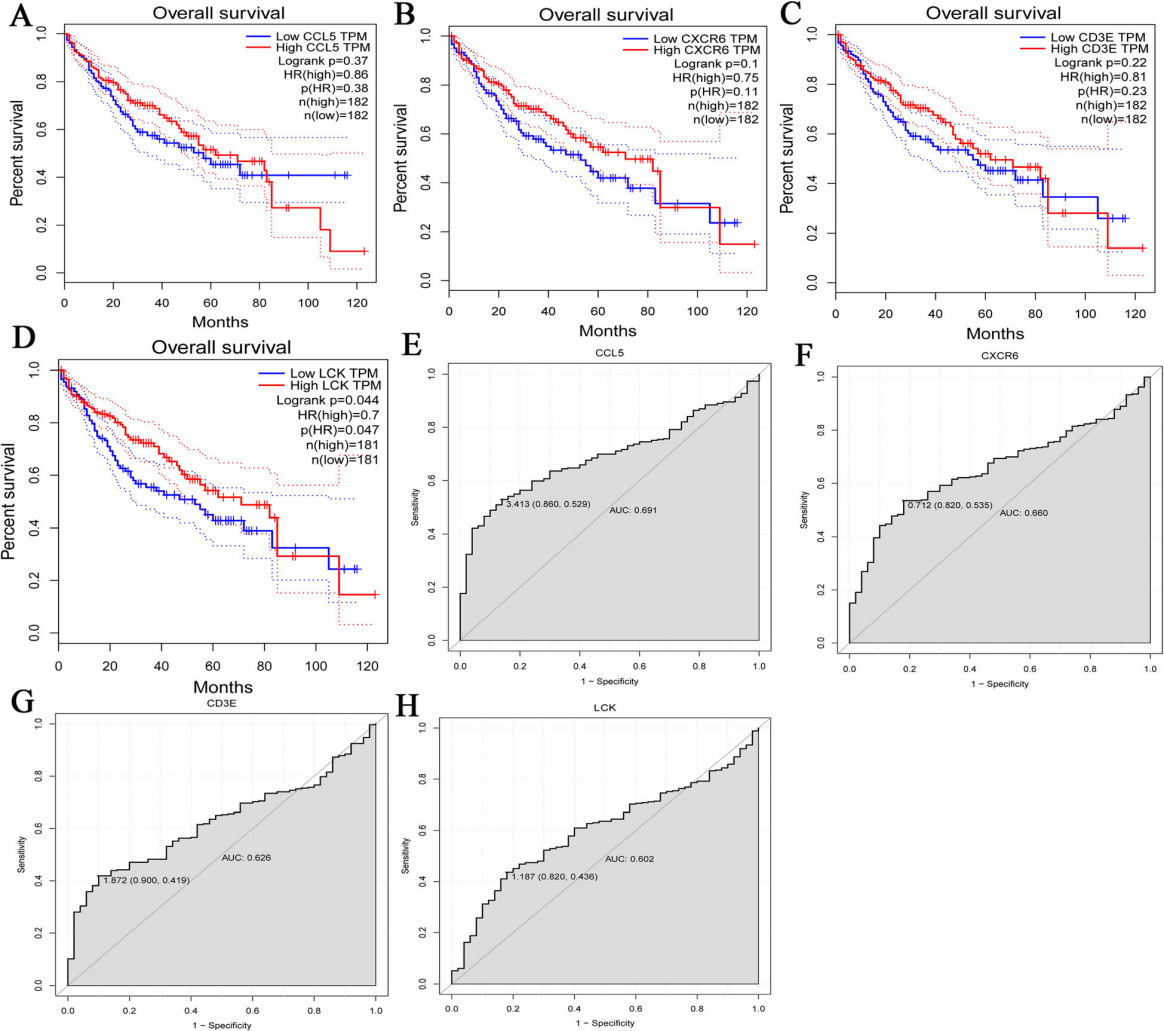
Prognostics and diagnostic analysis of CCL5, CXCR6, CD3E and LCK. **A** Survival curve of CCL5; **B** Survival curve for CXCR6; **C** Survival curve for CD3E; **D** Survival curve for LCK. *P* < 0.05 was considered to indicate a statistically significant difference. **E** ROC curve for CCL5; **F** ROC curve for CXCR6; **G** ROC curve for CD3E; **H** ROC curve for LCK. ROC, receiver operating characteristic; AUC: area under curve

**Fig. 11:**
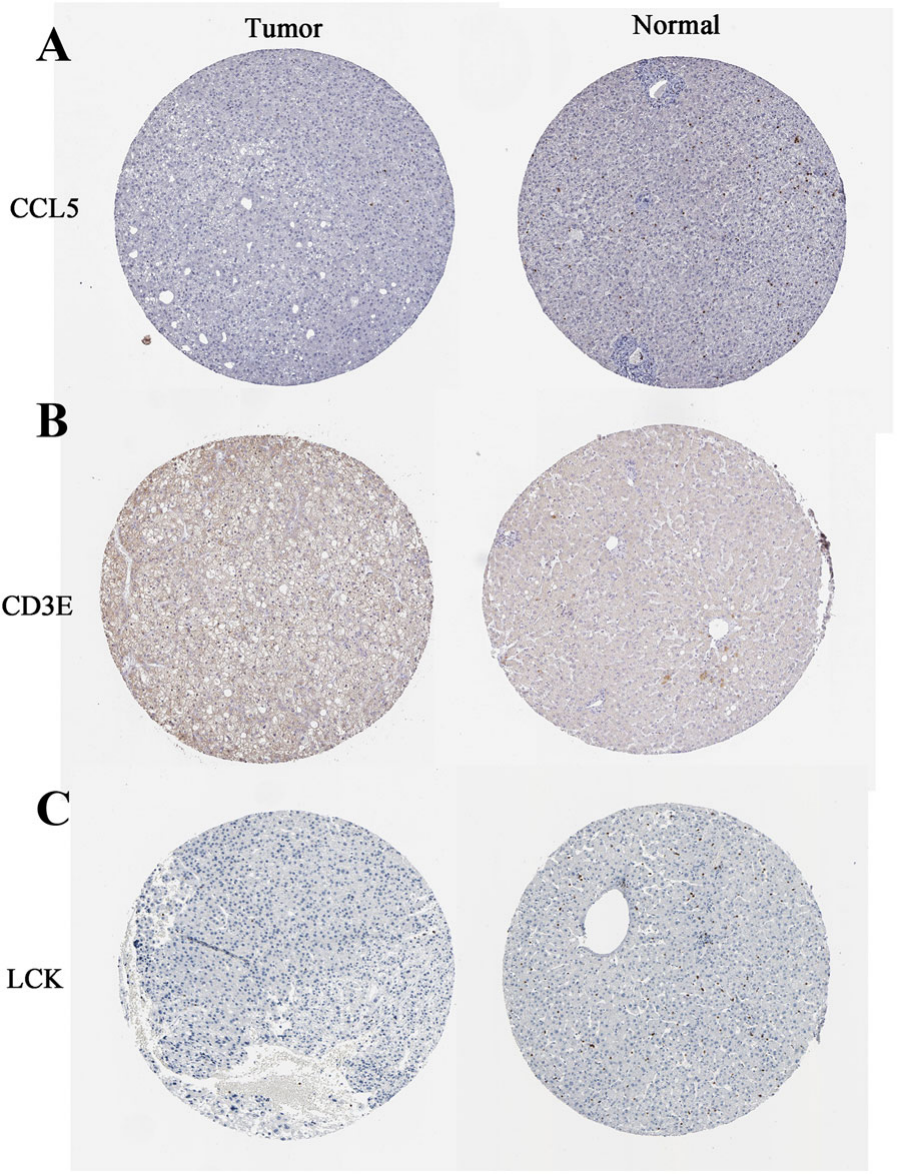
Immunohistochemical analysis of CCL5 (**A**), CD3E (**B**) and LCK (**C**). Normal and Tumor indicate adjacent non-tumor samples and HCC samples, respectively. HCC: hepatocellular carcinoma

Further analysis was performed to explore the impact of the 4 hub genes on survival and diagnosis. GEPIA was used for survival analysis of CCL5, CXCR6, CD3E and LCK (Fig. 10a-d). The present study demonstrated that survival prognosis of patients in the LCK low expression group was poor (*P* < 0.05) (Fig. 10d). Therefore, the present study used LCK as a prognostic biomarker for further analysis. Additionally, the TCGA data were used to analyze the diagnostic ability of CCL5, CXCR6, CD3E and LCK (Fig. 10a-d). In the receiver operating characteristic (ROC) curve analysis, the area under curve (AUC) of CCL5 and CXCR6 were 0.691 and 0.660, respectively (Fig. 10e, f), which is close to 0.7. This indicated that CCL5 and CXCR6 may be potential diagnostic gene biomarkers in HCC. CXCR6 was not found in online immunohistochemistry analysis, so only CCL5, CD3E and LCK were used for analysis. The immunohistochemistry results demonstrated that the staining intensity of CCL5, CD3E and LCK in HCC cancer tissues ended to be decreased compared with those in normal tissues (Fig. 11). This was consistent with the previous protein validation results (Fig. 8f).

## Discussion

HCC is a relatively common outcome of chronic liver infection or inflammation [27]. CD8^+^ T cells are essential effector cells in anti-tumor immunity [28]. Highly infiltrating CD8^+^ T cells are beneficial for tumor therapy of most tumors [29–31]. CD8^+^ T cells are key participants in the anti-tumor response of HCC [32]. Furthermore, intratumoral CD8^+^ T cells are associated with improved prognosis in patients with HCC after resection [33, 34]. In the present study, 9 hub genes (CCL5, CD2, CD3D, CD3E, CD3G, CTLA4, CXCR3, CXCR6 and LCK) related to the levels of CD8^+^ T cells infiltration were identified. Expression verification revealed that the expression levels of CCL5, CXCR6, CD3E and LCK decreased with the increase in pathological stage (Fig. 9). Notably, patients with low LCK expression had a poor survival prognosis (Fig. 10d). Therefore, LCK was considered as a potential prognostic marker and target.

LCK proto-oncogene, Src family tyrosine kinase (LCK) is one of the key molecules that regulate T cell function [35], and is involved in the immune response or lymphocyte activation [36]. LCK is a strong predictor of survival in high grade serous ovarian cancer (HGSOC), and immunoglobulin and B-cell related genes are highly expressed in samples with high LCK expression [37]. High LCK protein expression (a T-cell marker) is associated with improved patient survival in primary and/or metastatic melanoma [38]. In addition, LCK serves an essential role in cell migration and stemness gene expression [39]. In the present study, the expression levels of LCK exhibited a high correlation with the infiltration levels of CD8^+^ T cells (Fig. 5). Furthermore, LCK expression decreased with the increase of pathological stage (Fig. 9d). Notably, patients with low LCK expression had a poor survival prognosis (Fig. 10d). Therefore, it is hypothesized that LCK may be an important prognostic marker and immunotherapy target in HCC.

C-C motif chemokine ligand 5 (CCL5) is a chemokine produced by immune cells, and acts by binding to the corresponding receptor [40, 41]. Reduced CCL5 expression leads to desertification of tumor-infiltrating lymphocytes [42]. The disease-free survival of patients with early breast cancer with high CCL5 expression is improved compared with that of patients with low CCL5 expression [40]. Down-regulation of CCL5 has been detected in HCC tissue samples [43]. C-X-C motif chemokine receptor 6 (CXCR6) belongs to the CXC chemokine receptor family [44]. Previous studies regarding the effect of CXCR6 on liver cancer have revealed that after injection of diethylnitrosamine, the tumor load of CXCR6-deficient mice is markedly higher than that of wild-type mice, and tumor progression is increased. Furthermore, the number of natural killer T (NKT) and CD4^+^ T cells was decreased in the liver [45]. Notably, NKT and CD4^+^ T cells have been reported to promote senescence hepatocyte clearance to prevent hepatocarcinogenesis, and this process requires CXCR6 [45, 46]. CD3e molecule (CD3E) is a member of the CD3 complex, and deficiency can lead to immune deficiency [47]. Additionally, CD3E is a typical genetic marker associated with tumor-infiltrating lymphocytes [48]. Previous study has revealed that patients with head and neck squamous cell carcinoma (HNSCC) with low CD3E expression have a poor prognosis [49]. In the present study, the expression levels of CCL5, CXCR6 and CD3E exhibited a high correlation with the infiltration levels of CD8^+^ T cells (Fig. 5a, d), and the expression levels in HCC samples were lower than those that in adjacent non-tumor samples (Fig. 7 and Fig. 8). Furthermore, CCL5, CXCR6 and CD3E expression decreased with the increase of pathological stage (Fig. 9a-c). Therefore, it is hypothesized that CCL5, CXCR6 and CD3E may also be important regulatory genes and immunotherapeutic targets in HCC.

In summary, the present study suggested that CCL5, CXCR6, CD3E and LCK may be potential immunotherapy targets for HCC. LCK has been identified as a potential prognostic biomarker for immunotherapy in HCC. CCL5 and CXCR6 may be potential diagnostic gene biomarkers in HCC. The identification of these genes may be helpful in the development of early diagnosis and therapy of HCC. However, a certain degree of limitation exists in this experiment. To the best of our knowledge, the molecular mechanism of the identified genes in HCC is unclear, and further research is required.

## Conclusion

In the present study, 9 hub genes were identified using WGCNA and PPI network analysis. Furthermore, the expression levels of 9 genes were associated correlated with the infiltration levels of CD8^+^ T cells. In multiple dataset validations, CCL5, CXCR6, CD3E and LCK were identified to be down-regulated in cancer tissues. In addition, survival analysis demonstrated that patients with LCK low expression had a poor survival prognosis. The identification of CCL5, CXCR6, CD3E and LCK may be helpful in the development of early diagnosis and therapy of HCC. LCK may be a potential prognostic biomarker for immunotherapy in HCC.

## Supplementary Information

**Additional file 1: Supplementary Figure 1.** Differential expression of CD3E (A), CCL5 (B), CXCR6 (C) and LCK (D) in different tumor grades. A Kruskal-Wallis test was used to analyze the statistical significance among G1, G2 and G3-4. G: grade.

## Abbreviations

HCC: Hepatocellular carcinoma
WGCNA: Weighted gene co-expression network analysis
PPI: Protein-protein interaction
CD8+ T: CD8-positive T
TIICs: Tumor-infiltrating immune cells
ICF: Immune-mediated cancer field
GSEA: Gene Set Enrichment Analysis
TP53: Tumor protein p53
PD-1: Programmed cell death protein 1
RNA-seq: RNA sequencing
TCGA: The Cancer Genome Atlas
GEO: Gene Expression Omnibus
DAVID: Database for Annotation Visualization and Integrated Discovery
GO: Gene Ontology
KEGG: Kyoto Encyclopedia of Genes and Genomics
MM: Module connectivity
GS: Clinical trait relationship
STRING: Search Tool for the Retrieval of Interacting Genes/Proteins
TIMER: Tumor Immune Estimation Resource
TISIDB: Tumor Immune System Interactions Database
GEPIA: Gene Expression Profiling Interactive Analysis
LCK: LCK proto-oncogene, Src family tyrosine kinase
HGSOC: High grade serous ovarian cancer
CCL5: C-C motif chemokine ligand 5
CXCR6: C-X-C motif chemokine receptor 6
NKT: Natural killer T
CD3E: CD3e molecule
HNSCC: Head and neck squamous cell carcinoma

## References

[1] Hartke J, Johnson M, Ghabril M (2017). The diagnosis and treatment of hepatocellular carcinoma. Semin Diagn Pathol.

[2] Bray F, Ferlay J, Soerjomataram I, Siegel RL, Torre LA, Jemal A (2018). Global cancer statistics 2018: GLOBOCAN estimates of incidence and mortality worldwide for 36 cancers in 185 countries. CA Cancer J Clin.

[3] Marengo A, Rosso C, Bugianesi E (2016). Liver Cancer: connections with obesity, fatty liver, and cirrhosis. Annu Rev Med.

[4] de Martel C, Maucort-Boulch D, Plummer M, Franceschi S (2015). World-wide relative contribution of hepatitis B and C viruses in hepatocellular carcinoma. Hepatology.

[5] Chedid MF, Kruel CR, Pinto MA, Grezzana-Filho TJ, Leipnitz I, Kruel CD (2017). Hepatocellular carcinoma: diagnosis and operative management. Braz Arch Digest Surg.

[6] Shen Z, Li M, Bai S, Yang Q, Zhang F, Tang M, Guo J, Yang Z (2019). [Progress in immunotherapy for hepatocellular carcinoma]. Sheng wu gong cheng xue bao =. Chin J Biotechnol.

[7] Bremnes RM, Al-Shibli K, Donnem T, Sirera R, Al-Saad S, Andersen S (2011). The role of tumor-infiltrating immune cells and chronic inflammation at the tumor site on cancer development, progression, and prognosis: emphasis on non-small cell lung cancer. J Thorac Oncol.

[8] Choi Y, Kim JW (2017). Systemic inflammation is associated with the density of immune cells in the tumor microenvironment of gastric cancer. Gastric Cancer.

[9] Angell HK, Lee J, Kim KM, Kim K, Kim ST, Park SH, Kang WK, Sharpe A, Ogden J, Davenport A, Hodgson DR, Barrett JC, Kilgour E (2019). PD-L1 and immune infiltrates are differentially expressed in distinct subgroups of gastric cancer. Oncoimmunology.

[10] Hao X, Luo H, Krawczyk M, Wei W, Wang W, Wang J, Flagg K, Hou J, Zhang H, Yi S, Jafari M, Lin D, Chung C, Caughey BA, Li G, Dhar D, Shi W, Zheng L, Hou R, Zhu J, Zhao L, Fu X, Zhang E, Zhang C, Zhu JK, Karin M, Xu RH, Zhang K (2017). DNA methylation markers for diagnosis and prognosis of common cancers. Proc Natl Acad Sci U S A.

[11] Pagès F, Galon J, Dieu-Nosjean MC, Tartour E, Sautès-Fridman C, Fridman WH (2010). Immune infiltration in human tumors: a prognostic factor that should not be ignored. Oncogene.

[12] Zhang S, Zhang E, Long J, Hu Z, Peng J, Liu L, Tang F, Li L, Ouyang Y, Zeng Z (2019). Immune infiltration in renal cell carcinoma. Cancer Sci.

[13] Ye L, Li Y, Tang H, Liu W, Chen Y, Dai T, Liang R, Shi M, Yi S, Chen G, Yang Y (2019). CD8+CXCR5+T cells infiltrating hepatocellular carcinomas are activated and predictive of a better prognosis. Aging.

[14] Moeini A, Torrecilla S, Tovar V, Montironi C, Andreu-Oller C, Peix J (2019). An immune gene expression signature associated with development of human Hepatocellular Carcinoma identifies mice that respond to chemopreventive agents. Gastroenterology.

[15] Long J, Wang A, Bai Y, Lin J, Yang X, Wang D, Yang X, Jiang Y, Zhao H (2019). Development and validation of a TP53-associated immune prognostic model for hepatocellular carcinoma. EBioMedicine.

[16] Wu X, Gu Z, Chen Y, Chen B, Chen W, Weng L, Liu X (2019). Application of PD-1 blockade in Cancer immunotherapy. Comput Struct Biotechnol J.

[17] van IJzendoorn DG, Szuhai K (2019). Machine learning analysis of gene expression data reveals novel diagnostic and prognostic biomarkers and identifies therapeutic targets for soft tissue sarcomas. PLoS Comput Biol.

[18] Langfelder P, Horvath S (2008). WGCNA: an R package for weighted correlation network analysis. BMC Bioinformatics.

[19] Newman AM, Liu CL, Green MR (2015). Robust enumeration of cell subsets from tissue expression profiles. Nat Methods.

[20] Li R, Qu H, Wang S, Wei J, Zhang L, Ma R (2018). GDCRNATools: an R/Bioconductor package for integrative analysis of lncRNA, miRNA and mRNA data in GDC. Bioinformatics.

[21] Bosetti C, Turati F, La Vecchia C (2014). Hepatocellular carcinoma epidemiology. Best Pract Res Clin Gastroenterol.

[22] Edgar R, Domrachev M, Lash AE (2002). Gene expression omnibus: NCBI gene expression and hybridization array data repository. Nucleic Acids Res.

[23] Li T, Fan J, Wang B, Traugh N, Chen Q, Liu JS, Li B, Liu XS (2017). TIMER: a web server for comprehensive analysis of tumor-infiltrating immune cells. Cancer Res.

[24] Ru B, Wong CN, Tong Y, Zhong JY, Zhong SSW, Wu WC (2019). TISIDB: an integrated repository portal for tumor-immune system interactions. Bioinformatics.

[25] Ritchie ME, Phipson B, Wu D, Hu Y, Law CW, Shi W (2015). limma powers differential expression analyses for RNA-sequencing and microarray studies. Nucleic Acids Res.

[26] Tang Z, Li C, Kang B, Gao G, Li C, Zhang Z (2017). GEPIA: a web server for cancer and normal gene expression profiling and interactive analyses. Nucleic Acids Res.

[27] Piñeiro Fernández J, Luddy KA, Harmon C, O'Farrelly C (2019). Hepatic Tumor Microenvironments and effects on NK Cell Phenotype and function. Int J Mol Sci.

[28] Apetoh L, Smyth MJ, Drake CG, Abastado JP, Apte RN, Ayyoub M, Blay JY, Bonneville M, Butterfield LH, Caignard A, Castelli C, Cavallo F, Celis E, Chen L, Colombo MP, Comin-Anduix B, Coukos G, Dhodapkar MV, Dranoff G, Frazer IH, Fridman WH, Gabrilovich DI, Gilboa E, Gnjatic S, Jäger D, Kalinski P, Kaufman HL, Kiessling R, Kirkwood J, Knuth A, Liblau R, Lotze MT, Lugli E, Marincola F, Melero I, Melief CJ, Mempel TR, Mittendorf EA, Odun K, Overwijk WW, Palucka AK, Parmiani G, Ribas A, Romero P, Schreiber RD, Schuler G, Srivastava PK, Tartour E, Valmori D, van der Burg SH, van der Bruggen P, van den Eynde BJ, Wang E, Zou W, Whiteside TL, Speiser DE, Pardoll DM, Restifo NP, Anderson AC (2015). Consensus nomenclature for CD8(+) T cell phenotypes in cancer. Oncoimmunology.

[29] Galon J, Costes A, Sanchez-Cabo F, Kirilovsky A, Mlecnik B, Lagorce-Pagès C (2006). Type, density, and location of immune cells within human colorectal tumors predict clinical outcome. Science.

[30] Mahmoud SM, Paish EC, Powe DG, Macmillan RD, Grainge MJ, Lee AH (2011). Tumor-infiltrating CD8+ lymphocytes predict clinical outcome in breast cancer. J Clin Oncol.

[31] Sharma P, Shen Y, Wen S, Yamada S, Jungbluth AA, Gnjatic S, Bajorin DF, Reuter VE, Herr H, Old LJ, Sato E (2007). CD8 tumor-infiltrating lymphocytes are predictive of survival in muscle-invasive urothelial carcinoma. Proc Natl Acad Sci U S A.

[32] Li Z, Chen G, Cai Z, Dong X, Qiu L, Xu H (2019). Genomic and transcriptional Profiling of tumor infiltrated CD8(+) T cells revealed functional heterogeneity of antitumor immunity in hepatocellular carcinoma. Oncoimmunology.

[33] Huang CY, Wang Y, Luo GY, Han F, Li YQ, Zhou ZG (2017). Relationship Between PD-L1 Expression and CD8+ T-cell Immune Responses in Hepatocellular Carcinoma. J Immunother.

[34] Xu X, Tan Y, Qian Y, Xue W, Wang Y, Du J (2019). Clinicopathologic and prognostic significance of tumor-infiltrating CD8+ T cells in patients with hepatocellular carcinoma: a meta-analysis. Medicine.

[35] Bommhardt U, Schraven B, Simeoni L (2019). Beyond TCR signaling: emerging functions of Lck in Cancer and Immunotherapy. Int J Mol Sci.

[36] Katayama MLH, Vieira R, Andrade VP, Roela RA, Lima L, Kerr LM (2019). Stromal cell signature associated with response to Neoadjuvant Chemotherapy in locally advanced Breast cancer. Cells.

[37] Hinchcliff E, Paquette C, Roszik J, Kelting S, Stoler MH, Mok SC, Yeung TL, Zhang Q, Yates M, Peng W, Hwu P, Jazaeri A (2019). Lymphocyte-specific kinase expression is a prognostic indicator in ovarian cancer and correlates with a prominent B cell transcriptional signature. Cancer Immunol Immunother.

[38] Akbani R, Akdemir KC, Aksoy BA (2015). Genomic Classification of Cutaneous Melanoma. Cell.

[39] Zepecki JP, Snyder KM (2019). Regulation of human glioma cell migration, tumor growth, and stemness gene expression using a Lck targeted inhibitor. Oncogene.

[40] Fujimoto Y, Inoue N, Morimoto K, Watanabe T, Hirota S, Imamura M (2020). Significant association between high serum CCL5 levels and better disease-free survival of patients with early breast cancer. Cancer Sci.

[41] Lin J, Yu M, Xu X, Wang Y, Xing H, An J, Yang J, Tang C, Sun D, Zhu Y (2020). Identification of biomarkers related to CD8(+) T cell infiltration with gene co-expression network in clear cell renal cell carcinoma. Aging.

[42] Dangaj D, Bruand M, Grimm AJ, Ronet C, Barras D, Duttagupta PA (2019). Cooperation between Constitutive and Inducible Chemokines Enables T Cell Engraftment and Immune Attack in Solid Tumors. Cancer Cell.

[43] Fan W, Ye G (2018). Microarray analysis for the identification of specific proteins and functional modules involved in the process of hepatocellular carcinoma originating from cirrhotic liver. Mol Med Rep.

[44] Chang Y, Zhou L, Xu L, Fu Q, Yang Y, Lin Z (2017). High expression of CXC chemokine receptor 6 associates with poor prognosis in patients with clear cell renal cell carcinoma. Urol Oncol.

[45] Mossanen JC, Kohlhepp M, Wehr A, Krenkel O, Liepelt A, Roeth AA (2019). CXCR6 Inhibits Hepatocarcinogenesis by Promoting Natural Killer T- and CD4(+) T-Cell-Dependent Control of Senescence. Gastroenterology.

[46] Liepelt A, Wehr A, Kohlhepp M, Mossanen JC, Kreggenwinkel K, Denecke B, Costa IG, Luedde T, Trautwein C, Tacke F (2019). CXCR6 protects from inflammation and fibrosis in NEMO (LPC-KO) mice. Biochim et Biophys Acta Mol Basis Dis.

[47] Firtina S, Ng YY, Ng OH, Nepesov S, Yesilbas O, Kilercik M (2017). A novel pathogenic frameshift variant of CD3E gene in two T-B+ NK+ SCID patients from Turkey. Immunogenetics.

[48] Zhuang H, Zhang C, Hou B (2020). FAM83H overexpression predicts worse prognosis and correlates with less CD8(+) T cells infiltration and Ras-PI3K-Akt-mTOR signaling pathway in pancreatic cancer. Clin Transl Oncol.

[49] Lecerf C, Kamal M, Vacher S, Chemlali W, Schnitzler A, Morel C (2019). Immune gene expression in head and neck squamous cell carcinoma patients. Eur J Cancer.

