# Supplementary figures and images for: Identification of immune-infiltrating cell-related biomarkers in hepatocellular carcinoma based on gene co-expression network analysis
Source: Diagn Pathol. 2021 Jul 4;16:57. doi: 10.1186/s13000-021-01118-y (PMC8255019; doi:10.1186/s13000-021-01118-y)

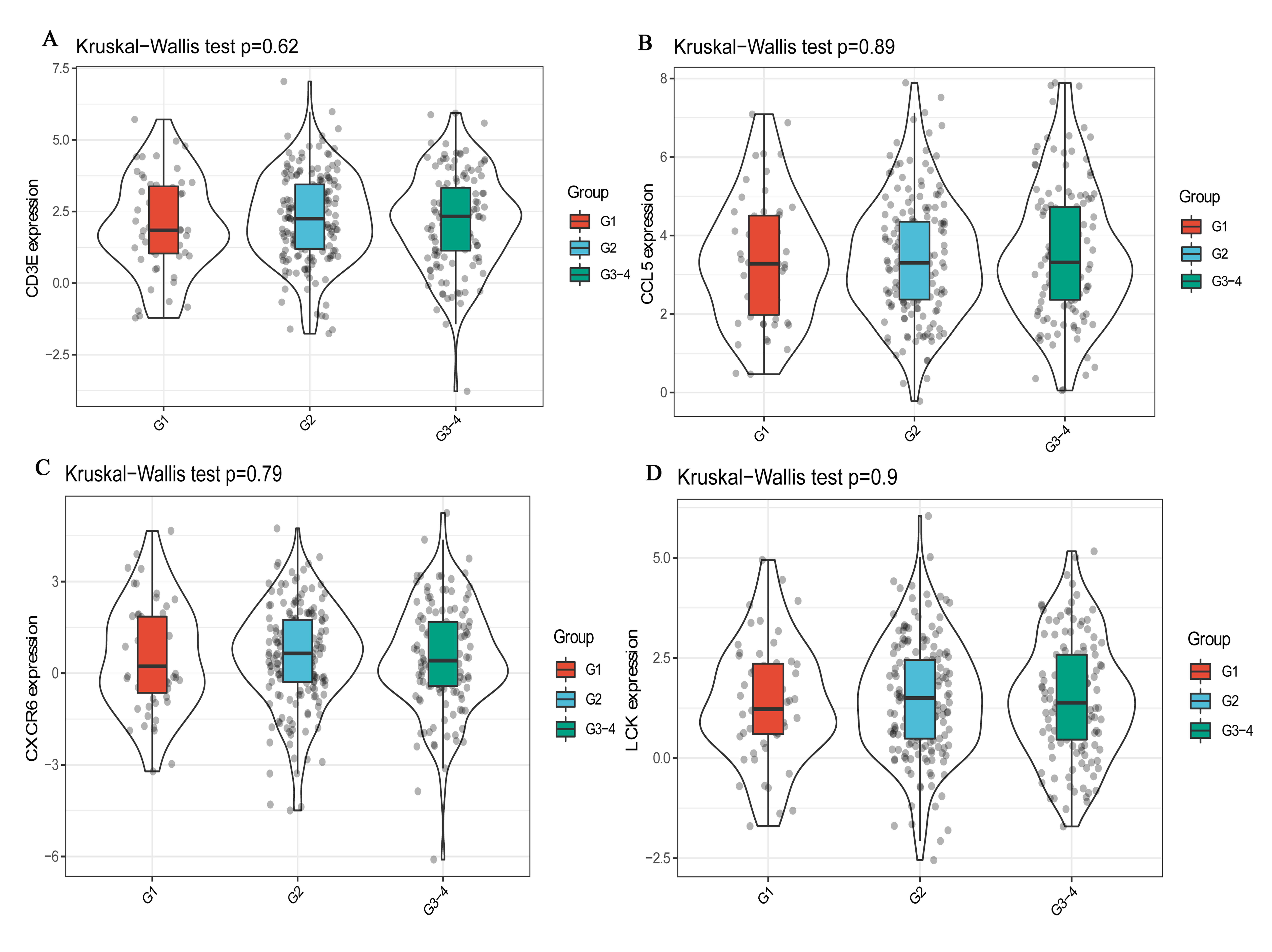

Supplement: Supplementary file 1 — Additional file 1: Supplementary Figure 1. Differential expression of CD3E (A), CCL5 (B), CXCR6 (C) and LCK (D) in different tumor grades. A Kruskal-Wallis test was used to analyze the statistical significance among G1, G2 and G3-4. G: grade. [file 13000_2021_1118_MOESM1_ESM.tif]
